# Theoretical Analysis of the Influence of Spacers on Salt Ion Transport in Electromembrane Systems Considering the Main Coupled Effects

**DOI:** 10.3390/membranes14010020

**Published:** 2024-01-10

**Authors:** Anna Kovalenko, Makhamet Urtenov, Vladimir Chekanov, Natalya Kandaurova

**Affiliations:** 1 Faculty of Computer Technologies and Applied Mathematics, Kuban State University, 350040 Krasnodar, Russia; urtenovmax@mail.ru; 2 Department of Digital Development, North-Caucasus Federal University, 355017 Stavropol, Russia; oranjejam@mail.ru; 3 Department of Information Technologies, MIREA-Russian Technological University, 355000 Stavropol, Russia; candaur18@yandex.ru

**Keywords:** electrodialysis, desalting, electroconvection, dissociation/recombination of water molecules, quasi-stationary state, Nernst–Planck–Poisson and Navier–Stokes equations system

## Abstract

This article considers a theoretical analysis of the influence of the main coupled effects and spacers on the transfer of salt ions in electromembrane systems (EMS) using a 2D mathematical model of the transfer process in a desalting channel with spacers based on boundary value problems for the coupled system of Nernst–Planck–Poisson and Navier–Stokes equations. The basic patterns of salt ion transport have been established, taking into account diffusion, electromigration, forced convection, electroconvection, dissociation/recombination reactions of water molecules, as well as spacers located inside the desalting channel. It has been shown that spacers and taking into account the dissociation/recombination reaction of water molecules significantly change both the formation and development of electroconvection. This article confirms the fact of the exaltation of the limiting current studied by Harkatz, where it is shown that the current (flux) of salt ions increases when the dissociation reaction begins by a certain value called the exaltation current, which is proportional to the flow of water dissociation products. A significant combined effect of electroconvection and dissociation/recombination reactions as well as the spacer system in the desalting channel on the transport of salt ions are shown. The complex, nonlinear, and non-stationary interaction of all the main effects of concentration polarization and spacers in the desalting channel are also considered in the work.

## 1. Introduction

Water stands out as humanity’s most vital resource and is crucial for all forms of life. However, at present, sustainable consumption is jeopardized in half of the world’s river basins [1]. The most precious form of water, clean drinking water, remains virtually inaccessible to around one billion people in developing countries.

The future scenario looks grim, not only due to climate change [2,3,4] but also because of the anticipated global population surge to 10 billion by 2050 and the rise in living standards accompanied by changing consumption patterns [5,6,7]. With a constrained supply of reliable drinking water, global water scarcity emerges as the foremost challenge for the world community [8]. The primary solution to address water scarcity involves economically and environmentally viable desalination methods [9,10].

Electrodialysis has gained global recognition as a water purification method with the potential to enhance the overall efficiency of the purification process [11].

The efficiency of electrodialysis depends strongly on the hydrodynamics of the process, as the advent of new high performance membranes on the world market removes the kinetic limitations associated with membranes and shifts the stage that determines the economic efficiency of desalination towards the liquid phase.

Studies in recent years show that there are two approaches that reduce the mass transfer limitations on the electrolyte solution side [12,13]. The first is the use of spacers with which the flow of the solution can be controlled. We have investigated this approach in our study [14,15]. Secondly, the use of electroconvection under intense current modes. However, in this case, such destructive processes as the long-known dissociation reaction of water molecules and the recently discovered space charge breakdown [16,17] arise, which lead to the problem of a joint study of all these processes and their complex influence on salt ions.

For the first time, the main regularities of salt ion transport in a desalting channel with spacers, electroconvection, and the dissociation/recombination reaction of water molecules and the interaction of these phenomena are established in this work theoretically, using the method of mathematical modeling.

### 1.1. Impact of the Dissociation/Recombination Reaction of Water Molecules on the Transport of Salt Ions

The papers [18,19] showed that the dissociation reaction of water molecules occurs intensively at current densities higher than the limiting one. Works by Sokirko and Harkatz [20,21] showed that the salt ion fluxes get exalted with the change of the electric field.

At the same time, in [22], it was first shown that the recombination reaction causes the appearance of a space charge region (SCR) in the central part of the desalination channel of the electrodialysis apparatus. The formation of SPZ in electrochemistry is associated with the presence of an interfacial boundary (solution/electrode or solution/ion exchange membrane). In [22], it was theoretically shown that H^+^ and OH^−^ ions move from the near-membrane regions to the central part of the channel, where a recombination zone appears, in which an SCR is formed from the terminal rate of the recombination reaction. The positive spatial charge of this SCR is due to the local excess of H^+^ ions moving away from the anion exchange membrane, and the negative spatial charge is due to the local excess of OH^−^ ions moving away from the anion exchange membrane and not having time to recombine due to the finiteness of the recombination reaction rate, although it is large. The dissociation reaction and formation of SCR in the recombination region affect the transport of salt ions as well as electroconvection. Moreover, a number of authors believe that the appearance of new charge carriers H^+^ and OH^−^ can lead to a decrease, or even disappearance of the space charge, which is the basis for other transport mechanisms, such as electroconvection.

Thus, accounting for the effect of the dissociation/recombination reaction of water molecules is important for understanding transport processes in electromembrane systems.

### 1.2. Effects of Electroconvection on Salt Ion Transport

During studies of super-limit transport, several interesting phenomena were discovered. One of them is electroconvection, the existence of which was proved experimentally and theoretically by Rubinstein and colleagues [23]. Electroconvection makes it possible to deliver “fresh” solutions from the center of the desalting channel to the membrane surface and to withdraw the desalinated solution from the membrane. It keeps a relatively high concentration of electrolytes at the membrane surface, which restrains the development of the process of generation of H^+^ and OH^−^ ions [24,25]. A smaller pH shift of the solution and its better mixing significantly reduce the rate of sludge formation in desalting and concentration chambers [26,27].

At present, electroconvection is the key mechanism of mass transfer intensification in EMS. Electroconvection also plays an important role in numerous microfluidic devices [28], such as electrokinetic micropumps [29], microconcentrators in analytical chemistry [30], shock electrodialysis [31,32], and others [33,34,35].

In the works of Rubinstein I. [36,37,38], Demekhin E.A., Kalaidin E.N. [39,40], the problems of occurrence and stability of electroconvection in micro- and nanofluidics in the absence of forced convection are investigated on the basis of mathematical modeling.

The works by Kwak R. [41], Pham V.S., Han J. [42], and others are devoted to the study of the patterns of transfer of salt ions taking into account electroconvection and the forced flow of solution. The peculiarity of electroconvection in the presence of forced convection is the presence of a different potential drop at which it occurs. In addition, electroconvection, which initially develops as a stable process, gradually becomes unstable with increasing potential drop, passing through a number of stages of bifurcations of electroconvective vortices and their interaction [43,44,45,46,47].

In [16,17], we first theoretically investigated the CVC characteristics for high current densities. The results show the presence of several modes of EMS operation associated with the development of electroconvective vortices arising near the cation exchange membrane (CEM) and subsequently near the anion exchange membrane (AEM). Then the vortices begin to actively interact, which leads to a breakdown of the space charge. Later vortices start to interact, which leads to space charge breakdown. This breakdown occurs when regions with positive and negative space charge detach from the membrane, move deep into the solution, meet, and their mutual neutralization occurs.

In [16,17], it was found that the effect of space charge breakdown restrains the development of electroconvective vortices, and leads to a decrease in the size and number of vortices in the breakdown region. This restrains a possible further increase in mass transfer due to electroconvection.

Thus, we have discovered a second limiting phenomenon at over limited current densities, which has never been analyzed before either experimentally or theoretically by simulations. The obtained results were published in [16,17].

### 1.3. Effects of Spacers on Salt Ion Transport

One of the effective methods to significantly increase the mass transport rate through membranes is the use of spacers, which allow the convective ion flux to be directed to the membrane surface [48,49,50].

Thus, the diffusion layer is significantly reduced in the channels with spacers and this leads to the current increase. This raises the question whether electroconvective vortices appear at high currents in such small diffusion layers. 

In a recent study, Kim et al. [51] evaluated the resulting hydrodynamics in the cross section of a spacer-filled channel in two-dimensional microscopic experiments and two-dimensional modeling. They found that off-center spacer placement increases the limiting current density due to the asymmetry of the electrolyte diffusion coefficients.

The paper [14,15] shows the results of the modeling of flow in desalting channels with and without spacers of different geometries. Modeling and comparative analysis of an empty electrodialysis desalting channel (without spacers) and four more desalting channels with spacers of different geometries were carried out. The influence of the shape, size, and position of spacers on the output current during the transport of NaCl salt ions in the desalting channel was investigated. In the articles [14,15] were developed 2D and 3D mathematical models of transport processes in a desalting channel with spacers. Diffusion, electromigration, forced convection, electroconvection, and membrane properties were taken into account in these models. However, the dissociation/recombination reaction of water molecules was not taken into account. It was shown that the location of spacers in the middle part of the channel leads to an insignificant increase in the CV characteristics plateau length, but at the same time increases the current output by 10–20%. The most promising spacer shapes, which lead to minimum resistance and maximum current increase compared to other spacer geometries, were found. At present, we are conducting further studies to analyze different shapes, sizes, angles, and locations of spacer geometries [14,15].

This paper is devoted to the theoretical analysis of the influence of the main coupled effects and spacers in electromembrane systems on salt ion transport using a 2D mathematical model of the transport process in a desalting channel with spacers taking into account diffusion, electromigration, forced convection, and electroconvection on the basis of boundary value problems for the coupled system of Nernst–Planck–Poisson and Navier–Stokes equations.

## 2. Materials and Methods

### 2.1. Geometry

**Figure 1:**
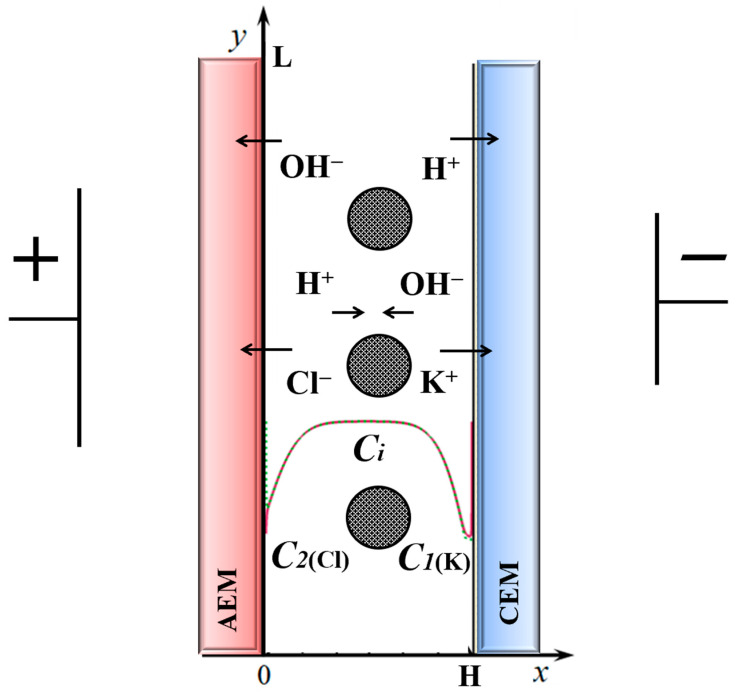
Diagram of a 2D desalination channel and simulated phenomena; the O*x* axis is directed across the channel, *x* = 0 corresponds to the boundary with the AEM, *x = H* to that with the CEM; the O*y* axis is directed along the channel, *y* = 0 refers to the channel inlet, *y = L*, to the outlet [34]. Non-conducting spacers are located in the middle of the channel.

We consider a desalting channel formed by AEM and CEM, through which a binary salt solution (e.g., KCl) flows with average velocity *V_0_* (Figure 1). The concentration of the solution at the inlet is *C_0_*, pH is 7. The potential difference between *x* = 0 and *x* = *H* increases linearly with time: φ(t,0,y)=d⋅t, where d is the sweep speed of the potential drop.

Figure 1 depicts the schematic diagram of the electrodialyzer desalting flow channel with integrated spacer system investigated in this paper. The channel is formed by ideally selective membranes, the anion-exchange membrane (AEM) and the cation-exchange membrane (CEM). The process of water splitting is shown schematically: at membranes, there is a process of dissociation of water molecules, so near AEM, H^+^ ions are formed, and at CEM, hydroxyl ions OH^−^, under the action of the electric field they move to the center of the channel, and there the process of recombination of water molecules takes place. Note that both of these processes are separated in space, dissociation is observed at the membranes, and recombination is observed in the center of the channel. In this study, we would like to investigate how the presence of a spacer system in the electrodialyzer desalting flow channel would affect the salt ion transport process considering the electroconvection and dissociation/recombination reaction of water molecules.

### 2.2. Equations

The non-stationary transfer of salt ions for a 1:1 electrolyte, taking into account the space charge and the dissociation/recombination reaction, is described by the following 2D system of equations:

(1) $$\frac{\partial C_{i}}{\partial t}=-div\;{\vec{j}}_{i}+R_{i},\;i=1,\ldots ,4$$

(2) $${\vec{j}}_{i}=-z_{i}\frac{F}{RT}D_{i}C_{i}\nabla \varphi -D_{i}\nabla C_{i}+C_{i}\vec{V},\;i=1,\ldots ,4$$

(3) $$\Delta \varphi =-\frac{\rho }{\varepsilon_{r}}$$

(4) $$R_{1}=R_{2}=0,\;R_{3}=R_{4}=k_{d}C_{H_{2}O}-k_{r}C_{3}C_{4}=k_{r}(K_{w}-C_{3}C_{4})$$

$$z_{1}=1,\;z_{2}=-1,\;z_{3}=1,\;z_{4}=-1$$

(5) $$I=F\sum_{i=1}^{4}z_{i}{\vec{j}}_{i}$$

(6) $$\frac{\partial \vec{V}}{\partial t}+\left(\vec{V}\nabla \right)\vec{V}=-\frac{1}{\rho_{0}}\nabla P+\nu \Delta \vec{V}+\frac{1}{\rho_{0}}\vec{f},$$

(7) $$\nabla \cdot \vec{V}=0$$

(8) $$\rho =F\sum_{i=1}^{4}z_{i}C_{i}$$

Here, Equation (1) is the species conservation equation; Equation (2) is the Nernst–Planck equation written for the flux densities of potassium ($K^{+},\;i=1$), chloride ($Cl^{-},\;i=2$), hydrogen ($H^{+},\;i=3\;$), and hydroxide ($OH^{-},\;i=4$) ions; Equation (3) is the Poisson equation relating the electric potential and space charge density (8). Equations (1)–(8) are described in detail in our previous works, for example, in [22]. 

### 2.3. Boundary and Initial Conditions


(9) $${-\vec{n}\cdot \left(-\frac{F}{RT}C_{1}D_{1}\nabla \varphi -D_{1}\nabla C_{1}\right)|}_{x=0}=0$$

(10) $$C_{2}(t,0,y)=C_{2a}$$

(11) $${-\vec{n}\cdot \left(-\frac{F}{RT}C_{3}D_{3}\nabla \varphi -D_{3}\nabla C_{3}\right)|}_{x=0}=0$$

(12) $$-\vec{n}\cdot \nabla C_{4}(t,0,y)=0$$

(13) $$\vec{V}(t,0,y)=0$$

(14) φ(t,0,y)=d⋅t

(15) $$C_{1}(t,H,y)=C_{1c}$$

(16) $$-\vec{n}\cdot {\left(\frac{F}{RT}C_{2}D_{2}\nabla \varphi -D_{2}\nabla C_{2}\right)|}_{x=H}=0$$

(17) $$-\vec{n}\cdot \nabla C_{3}(t,H,y)=0$$

(18) $$-\vec{n}\cdot {\left(\frac{F}{RT}C_{4}D_{4}\nabla \varphi -D_{4}\nabla C_{4}\right)|}_{x=H}=0$$

(19) $$\vec{V}(t,H,y)=0$$

(20) φ(t,H,y)=0

(21) $$C_{i}(t,x,0)=C_{i,0},\;\;i=1,\ldots ,4,$$

(22) $$\sum_{i=1}^{4}z_{i}C_{i}(t,x,0)=0$$

(23) $${V_{x}|}_{y=0}=0$$

(24) $${V_{y}|}_{y=0}=6V_{0}\frac{x}{H}\left(1-\frac{x}{H}\right)$$

(25) φ(t,x,0)=0

(26) $$-\vec{n}\cdot \nabla \varphi (t,x,L)=0$$

(27) $${-\vec{n}\cdot \nabla V_{y}|}_{y=L}=0$$

(28) $${-\vec{n}\cdot \nabla V_{x}|}_{y=L}=0$$

The extended SCR under the electric force causes electroconvection, which affects the velocity of the solution, described by the Navier–Stokes equation. The change in velocity affects the ionic fluxes, which are determined by the NPP equations.

The boundary conditions (9)–(28) assume continuity of fluxes across the solution/membrane interfaces and selectivity of the membrane surface. In addition, the concentrations of counterions are given as parameters C_im_ on the membrane surface. The sticking condition at *x* = 0 and *x* = *H* is used, (13) and (19). 

Since only non-catalytic water dissociation/recombination reactions are considered, it is assumed that there is no injection of hydrogen and hydroxyl ions from the membrane surface into the solution: ${j_{3,x}|}_{x=0}=j_{3a}=0$, ${j_{4,x}|}_{x=H}=j_{4c}=0$.

The potentiodynamic regime when the potential difference increases with some sweep rate *d* is considered: φ(t,0,y)=d⋅t.

We investigated the most significant processes in the desalting channel, which are formed by COM and AOM exactly in the order in which they are shown in Figure 1. In this case, the membranes are considered to be ideally selective, which is reflected in the boundary conditions (9)–(11), (16)–(18).

It is also believed that the exchange capacities of the membranes are known and the conditions (10)–(15) are determined by them. In addition, the membrane surfaces are considered to be equipotential, which is reflected in the boundary conditions (14)–(20).

The initial conditions are assumed to be consistent with the boundary conditions:

(29) $$C_{1}(0,x,y)=C_{2}(0,x,y)=C_{0}$$

(30) $$C_{3}(0,x,y)=C_{4}(0,x,y)=\sqrt{K_{w}}$$

(31) φ(0,x,y)=0

(32) $$V_{y}(0,x,y)=6V_{0}\frac{x}{H}\left(1-\frac{x}{H}\right)$$

(33) $$V_{x}(0,x,y)=0$$

Equation system (1)–(33) is a boundary value problem for a system of nonlinear partial differential equations of the second order. The numerical solution of such boundary value problems causes significant difficulties, since such problems are multidimensional and contain a large number of variables. In addition, when solving the problems, it is necessary to take into account the change of variables at different scales—from several nanometers in the volume of the electric double layer at the interfacial boundary to several centimeters along the length of the channel formed by two membranes.

### 2.4. Numerical Solution Algorithm

The numerical solution is based on the finite element method and splitting the problem at each current time layer into hydrodynamic (Navier–Stokes equation) and electrochemical (Nernst–Planck–Poisson) problems following by successive alternating solutions to convergence with a given accuracy. We successfully applied this method to solve a number of electrochemical problems [16].

## 3. Results

Many calculations at different initial parameters were carried out during this study. Below are the results, for specificity, at the following values: the concentration of the initial solution *C*_0_ is taken equal to 0.01 mol∙m^−3^; the intermembrane distance *H* is 0.5 mm; the channel length *L* = 2 mm; the liquid velocity at the inlet *V*_0_ = 0.1 mm∙s^−1^. 

As for *d*, its value is chosen in such a way as to ensure quasi-stationarity of the ED process, namely, when the sweep speed is set less than this value, the shape of the current–voltage curve (CVC) does not change. Below in calculations *d* = 10 mV/s.

### Formula for Calculating the CV Characteristics

To calculate the theoretical current–voltage characteristic for salt ions, we use the formula [47], which allows us to find it numerically stable with respect to rounding errors:

$$i_{av}(t)=\frac{1}{S}\int_{0}^{H}\int_{0}^{L}I_{x}(t,s,y)dydx,$$

where *S* = *HL* in the case of an empty channel and *S* = *HL* – *S*_0_, where *S*_0_ is the area of non-conducting spacers. Note that although these formulas do not explicitly include the concentrations of H^+^ and OH^−^ ions, they depend implicitly on them, since the potential and the flow rate of the solution depend on the dissociation/recombination reaction, i.e., on *C*_3_, *C*_4_. As a consequence, in the future it is necessary to compare the current–voltage characteristics calculated both with and without taking into account this reaction, obtained in [47].

To go to the dimensionless form, we will use the limiting diffusion current according to Leveque:

(34) $$i_{\lim}=\frac{FDC_{0}}{H\left(T_{1}-t_{1}\right)}\left[1.47{\left(\frac{H^{2}V_{0}}{LD}\right)}^{1/3}-0.2\right],\;\;\;T_{1}=1,\;\;t_{1}=0.5$$

where *D* is the diffusion coefficient of the electrolyte, *T*_1_, *t*_1_ are the transfer numbers of salt ions in the solution and membrane.

## 4. Fundamental Regularities of Transfer

### 4.1. Methodology of Theoretical Study

The theoretical study methodology consists of numerical investigation of different mathematical models. All these models consider diffusion, electromigration, convective transport, and electroconvection, and differ in that they take into account or do not take into account the influence of spacers and dissociation/recombination reactions of water molecules. Thus, to theoretically investigate the fundamental ion transport patterns of a binary 1:1 salt, we perform a comparative analysis of the different characteristics for four different cases:

1. Spacers are present and the dissociation/recombination reaction of water molecules is considered;
2. Spacers are absent and the dissociation/recombination reaction of water molecules is considered;
3. Spacers are present and the reaction of dissociation/recombination of water molecules is not considered;
4. Spacers are absent and the reaction of dissociation/recombination of water molecules is not considered.

**Figure 2:**
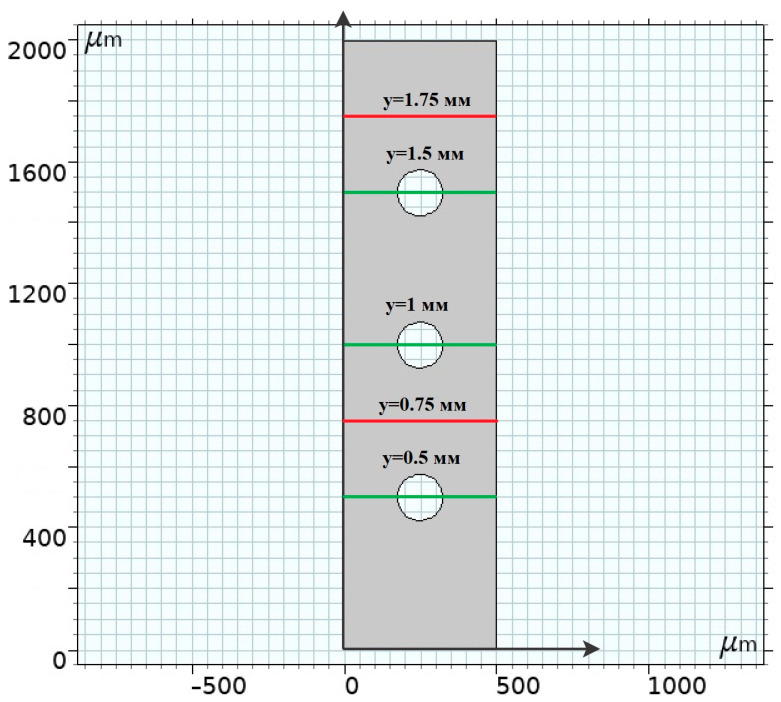
Sections of the channel where the desired functions are investigated. Cross sections across non-conducting spacers are highlighted in green in the section passing through the center of the middle spacers y = 0.50, 1.00, 1.50 mm. The lines that run across the channel and do not affect the spacers are highlighted in red: Between the first and second spacers in the front part of the cell y = 0.75 mm; Between the third spacer near the exit from the channel y = 1.75 mm.

In this case, along with two-dimensional plots, we will study in more detail the transport process in different sections of the channel (Figure 2):

### 4.2. Basic Regularities of Spatial Charge Variation

**Figure 3:**
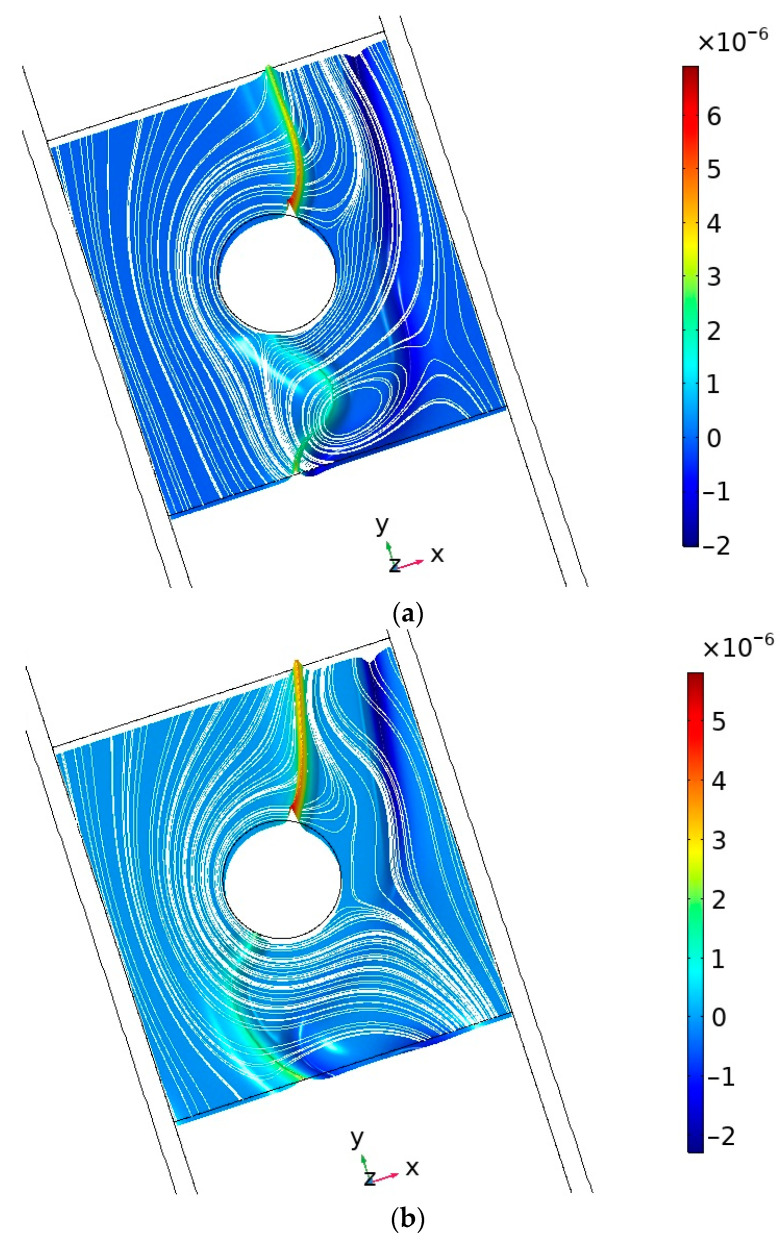
General view of the cross section of normalized space charge density without considering space charge at ion exchange membranes (highlighted) solution current lines (white lines) at times (**a**) 130 s and (**b**) 131 s.

Figure 3 shows general cross-sectional views of the normalized spatial charge density *ρ* in the vicinity of the spacer without considering the spatial charge at ion exchange membranes, which shows the complex and non-stationary interaction of solution flow and spatial charge distribution (Figure 3).

**Figure 4:**
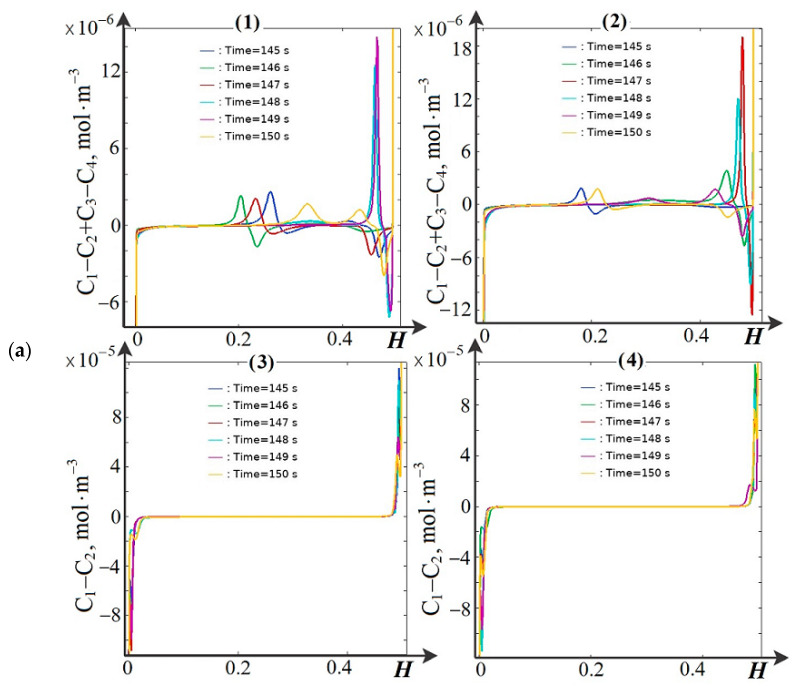

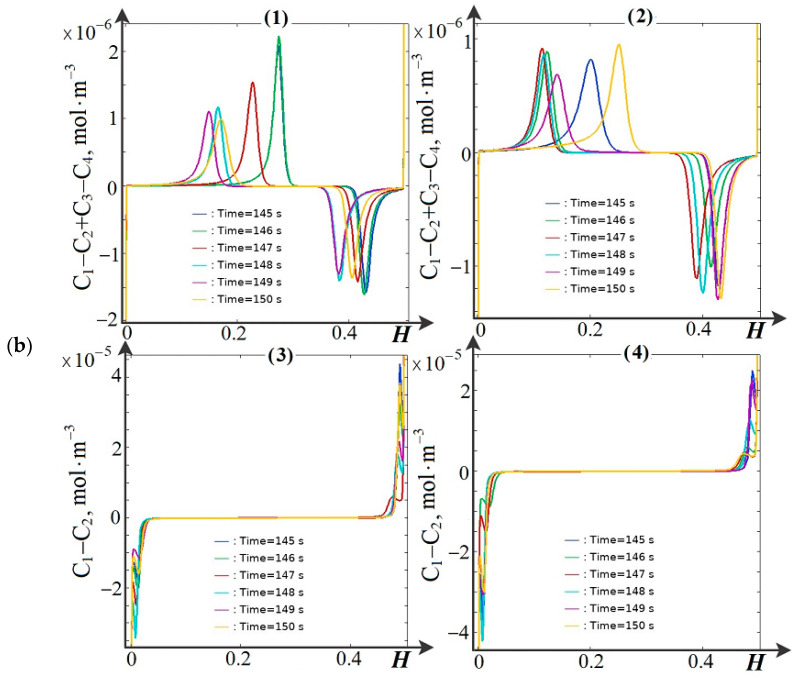
Spatial charge densities normalized by Faraday number in channel cross sections at (**a**) *y* = 0.75 mm, (**b**) *y* = 1.75 mm, for four electrodialyzer desalting channels at different time points in which: (**1**) spacers are present and the dissociation/recombination reaction of water molecules is considered; (**2**) spacers are absent and the dissociation/recombination reaction of water molecules considered; (**3**) spacers are present and the dissociation/recombination reaction of water molecules is not considered; (**4**) spacers are absent and the dissociation/recombination reaction of water molecules is not considered. *T* = 145 s, *d* = 1.45 V; *t* = 146 s, *d* = 1.46 V; *t* = 147 s, *d* = 1.47 V; *t* = 148 s, *d* = 1.48 V; *t* = 149 s, *d* = 1.49 V; *t* = 150 s, *d* = 1.5 V.

For a more detailed analysis, let us consider the cross-sectional distribution of the normalized space charge density. Figure 4 shows the cross-sectional views of the normalized space charge density *ρ*/*F* at *y* = 0.75 mm and *y* = 1.75 mm at different moments of time (*t* = 145 s, 146 s, 147 s, 148 s, 149 s, 150 s). It can be seen that if spacers are absent and the dissociation/recombination reaction is not taken into account, the standard picture is observed—the spatial charge is concentrated near the ion exchange membranes (Figure 4(a4,b4)), and the electroneutrality region is located in the middle of the channel. The presence of spacers (Figure 4(a3,b3)) does not change this picture, since they are non-conductive. A completely different picture is observed when the dissociation/recombination reaction of water molecules is taken into account. In the first case, an additional internal double electric layer (DEL) appears in the middle part of the channel, in addition to the space charge region (SCR) at the membranes. The reasons for the appearance of the internal double electric layer are explained above and discussed in detail in [16]. The space charge profiles at *y* = 0.75 mm are an order of magnitude larger than at *y* = 1.75 mm and qualitatively repeat each other. If a non-conducting spacer is located in the middle part of the channel (Figure 4(a2,b2)), the inner DEL is either shifted (to the left or to the right—depending on the type of electrolyte) away from the spacer (Figure 4(b2)) or split into two inner DECs (Figure 3a,b and Figure 4(a2)).

In section *y* = 1.75 mm, the profiles of normalized spatial charge correspond to the profiles in section *y* = 0.75 mm obtained at higher values of potential drop or time. This is explained by the fact that desalination of the solution occurs during the transition from the *y* = 0.75 mm cross section to the *y* = 1.75 mm cross section. It is known that the more desalinated the solution, the stronger the effects associated with space charge formation. Figure 4a shows that at the beginning of the channel, the positive and negative branches of the internal volume charge are located next to each other, but they diverge more and more as the solution moves towards the channel outlet. This is due to the fact that in the middle part of the channel a depleted (desalted) region of the solution is formed (see Figure 3), similar to that in shock electrodialysis [16]. Large changes in the spatial charge distribution occur with increasing time—these can be seen by comparing Figure 4a,b. These changes are due to electroconvection, which occurs due to the effect of the electric force on the regions of spatial charges occurring both on the membranes and in the solution volume.

### 4.3. Main Regularities of the Influence of the Dissociation/Recombination Reaction of Water Molecules on Salt Ion Transport

**Figure 5:**
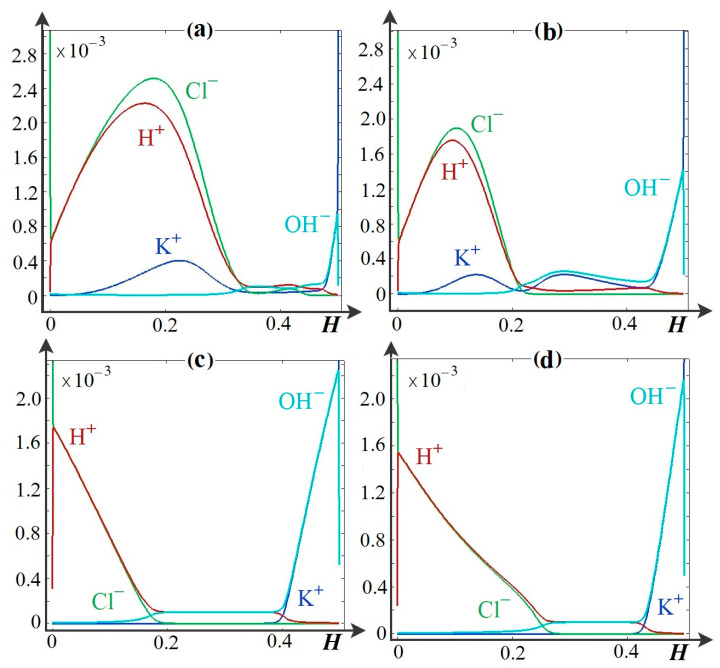
Concentrations of K^+^, Cl^−^, H^+^, and OH^−^ ions in channel cross sections at (**a**) *y* = 0.75 mm, spacers are present and dissociation/recombination reaction of water molecules is taken into account, (**b**) *y* = 0.75 mm, spacers are absent, dissociation/recombination reaction of water molecules is taken into account, (**c**) *y* = 1. 75 mm, spacers are present and dissociation/recombination reaction of water molecules is taken into account, (**d**) *y* = 1.75 mm, spacers are absent, dissociation/recombination reaction of water molecules is taken into account, at *t* = 150 s, *d* = 1.5 V.

**Figure 6:**
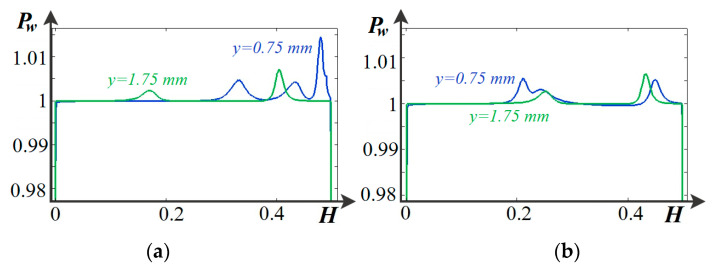
Equilibrium function *P_w_* in channel cross sections at (**a**) *y* = 0.75 mm, *y* = 1.75 mm, spacers are present and water molecule dissociation/recombination reaction is accounted for, (**b**) *y* = 0.75 mm, *y* = 1.75 mm, spacers are absent, water molecule dissociation/recombination reaction is accounted for, at *t* = 150 s, *d* = 1.5 V.

Numerical calculations show that several qualitatively different periods are distinguished as the potential drop increases. The first period in the range of low potential drops corresponds to the usual mode of electrodialysis with spacers [15,16,17]. Later on, there is both a decrease in the concentration of salt ions and an increase in the concentration of H^+^ and OH^−^ ions, due to the non-catalytic dissociation of water molecules (Figure 5). When these concentrations become comparable, the second period begins. It is due to the fact that dissociation begins to prevail over recombination, i.e., in all areas of the solution, salt ions predominate (Figure 5), and the space charge is concentrated near the ion exchange membranes. That is why the function Pw is less than unity only near the surfaces of AEM and CEM (Figure 6). Where *P_w_* = *C*_3_*C*_4_/*K_w_* is the reduced ion-product of water. In the region where *P_w_* < 1, dissociation prevails over recombination, and vice versa, recombination dominates when *P_w_* > 1; *P_w_* = 1 signifies that the rates of dissociation and recombination processes are the same, and H^+^ and OH^−^ ions are in equilibrium with water molecules.

### 4.4. Basic Regularities of Occurrence and Development of Electroconvection

**Figure 7:**
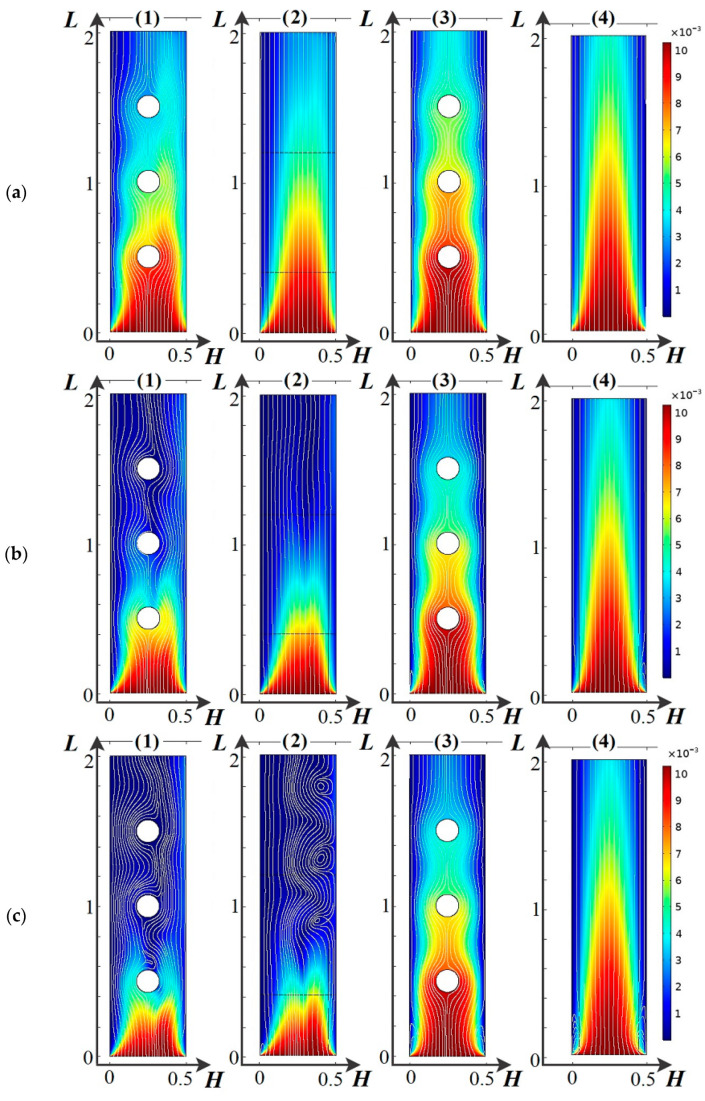

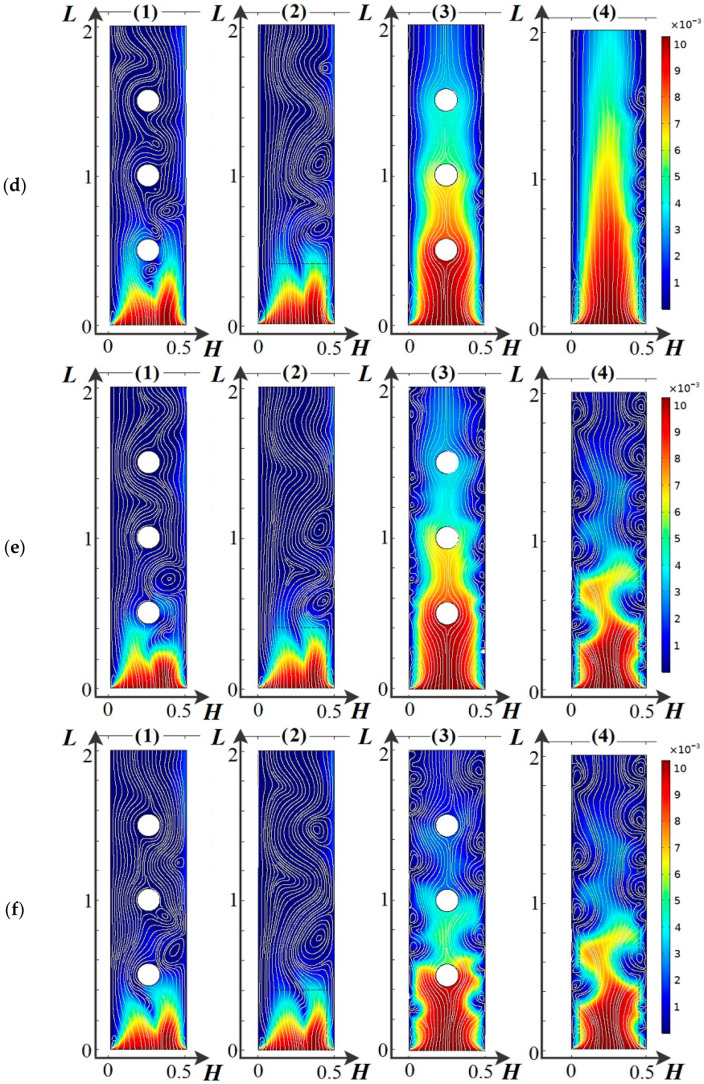

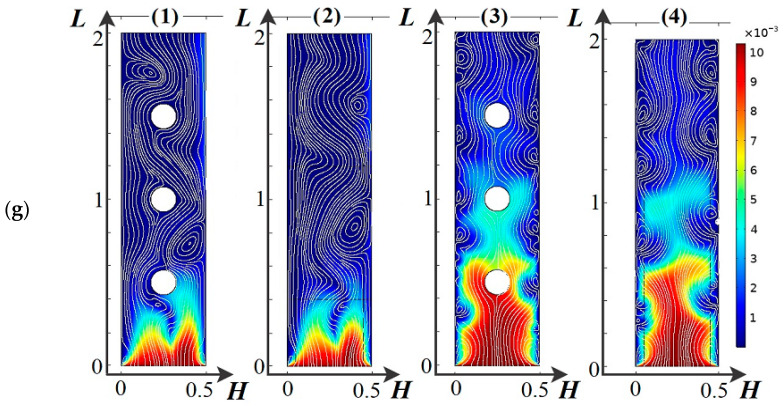
Cation concentration surfaces (color scale) and fluid stream lines (white) for four electrodialyzer desalting channels at different time points in which: (**1**) spacers are present and the dissociation/recombination reaction of water molecules is considered, (**2**) spacers are absent and the dissociation/recombination reaction of water molecules is considered, (**3**) spacers are present and the dissociation/recombination reaction of water molecules is not considered, (**4**) spacers are absent and the dissociation/recombination reaction of water molecules is not considered. (**a**) *t* = 30 s, *d* = 0.3 V; (**b**) *t* = 60 s, *d* = 0.6 V; (**c**) *t* = 90 s, *d* = 0.9 V; (**d**) *t* = 120 s, *d* = 1.2 V; (**e**) *t* = 135 s, *d* = 1.35 V; (**f**) *t* = 149 s, *d* = 1.49 V; (**g**) *t* = 150 s, *d* = 1.5 V.

Let us consider cation concentration surfaces (color scale) and fluid stream lines (white color) for four electrodialyzer desalting channels at different moments of time (Figure 7), in which: (1) spacers are present and the dissociation/recombination reaction of water molecules is considered, (2) spacers are absent and the dissociation/recombination reaction of water molecules is considered, (3) spacers are present and the dissociation/recombination reaction of water molecules is not considered, (4) spacers are absent and the dissociation/recombination reaction of water molecules is not considered.

Figure 7a–g show the distribution of K^+^ concentration and liquid current line in the ED desalination channel at different times since the beginning of the linear VAage sweep ((a) *t* = 30 s, *d* = 0.3 V; (b) *t* = 60 s, *d* = 0.6 V; (c) *t* = 90 s, *d* = 0.9 V; (d) *t* = 120 s, *d* = 1.2 V; (e) *t* = 135 s, *d* = 1.35 V; (f) *t* = 149 s, *d* = 1.49 V; (g) *t* = 150 s, *d* = 1.5 V). The results are presented for four scenarios of the desalting process: (1) considering water dissociation/recombination reactions and spacers, (2) considering water dissociation/recombination reactions but without spacers, (3) not considering water dissociation/recombination reactions but with spacers, (4) not considering water dissociation/recombination reactions and without spacers. 

Let us consider the formation and development of electroconvection [52,53,54] in the desalting channel of the electrodialyzer (Figure 7a–g). Considering the dissociation/recombination reaction of water molecules significantly changes both the formation and development of electroconvection (Figure 7a–g). 

As mentioned above and shown in Figure 7, initially the concentration of salt ions decreases faster under the action of dissociation/recombination reaction (Figure 7a–g). At the same time, in the downstream solution, the fluid current lines are curved in the middle part of the channel due to the presence of a double electric layer in this region (Figure 7c–g). Further, these curvatures increase, which increases the inflow of solution from the middle part of the channel in the direction of ion exchange membranes. As a result, a desalting region is formed in the middle part of the channel; it is visible at *t* = 90 s (0.9 V) (Figure 7c) and particularly well at *t* = 150 s (1.5 V) (Figure 7f). At the same time, no curvature of the solution current lines can be seen (Figure 7c), where the effect of the dissociation/recombination reaction is not taken into account. Under the action of the dissociation/recombination of water molecules, electroconvection starts at the cation exchange membrane from approximately *t* = 90 s (0.9 V). At time *t* = 120 s (1.2 V), the developed electroconvective vortices are already clearly visible (Figure 7d). At the same time, in the absence of dissociation/recombination reactions, electroconvective vortices appear (Figure 7(d3,d4)), with the presence of the spacer system slowing down their development for a few seconds. However, with time, the spatial charges at the ion-exchange membranes begin to be suppressed by H^+^ and OH^−^ ions arising from the dissociation reaction and having opposite signs to their respective spatial charges. Therefore, the electroconvective vortices [55] decrease, as can be seen in the comparison (Figure 7f,g).

Thus, the dissociation/recombination reaction of water molecules leads to a faster desalting of the solution initially and thus an earlier onset of electroconvection. However, with time, electroconvection begins to be inhibited by new H^+^ and OH^−^ charge carriers. In addition, the spacers provide better mixing of the solution and, consequently, the influence of the dissociation/recombination reaction of water molecules decreases, due to the lengthening of the first period (n.4.2).

### 4.5. Main Patterns of Change in the Current–Voltage Characteristic

**Figure 8:**
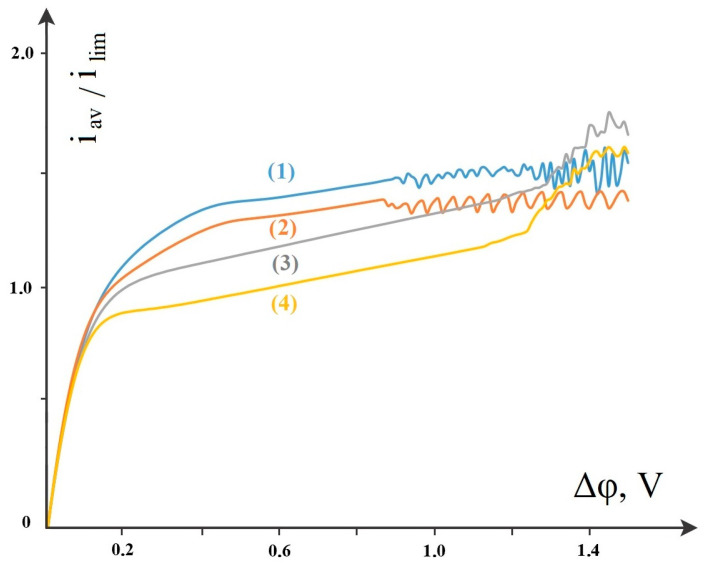
Comparisons of CVCs across salt ions where: (1) spacers are present and the dissociation/recombination reaction of water molecules is considered, (2) spacers are absent and the dissociation/recombination reaction of water molecules is considered, (3) spacers are present and the dissociation/recombination reaction of water molecules is not considered, (4) spacers are absent and the dissociation/recombination reaction of water molecules is not considered.

There are no electroconvective vortices within the range of potential drop change up to 0.9 V. As follows from Figure 8, the dissociation/recombination reaction increases the CVC for salt ions up to 1.25 V (which corresponds to about 2*I_lim_*). This is due to the known fact of the exaltation of the limiting current investigated by Harkatz et al. in [20,21], where it was shown that the current (flux) of salt ions increases when the dissociation reaction starts by some value called the exaltation current, which is proportional to the flux of water dissociation products [20,21]. In Figure 8, the difference between the upper (1–2) CVCs considering dissociation\recombination of water molecules and the lower ones (3–4) without considering this reaction is the exaltation current normalized to the limiting diffusion current. An increase in the cation fluxes, in turn, leads to an increase in the current density on salt ions, and hence to a decrease in the cation concentration.

Electroconvective vortices start, as shown above, at 0.9 V, which leads to a fluctuating CVC (Figure 8, curves 1–2) with a small, almost linear increasing trend. As soon as electroconvection starts in the models without taking into account the dissociation/recombination reaction (3–4), their corresponding CVCs become much larger than in the models taking this reaction into account. This is due to the fact that the spatial charge of the near-membrane regions due to the non-catalytic dissociation reaction becomes much smaller, and, consequently, the electroconvective vortices will be smaller. Because of this, the delivery of fresh solution from the depth of the channel in the problems without taking dissociation into account is much larger than in the problems with taking it into account. Accordingly, the fluxes and currents will be larger.

## 5. Conclusions

A theoretical study of the influence of spacers on the transport of salt ions with regard to the dissociation/recombination reaction of water molecules and electroconvection has been carried out in this paper. A complex, nonlinear, contradictory, nonstationary interaction and the mutual influence of spacers, dissociation/recombination reaction, and electroconvection between each other are shown. The results show that spacers slightly suppress water splitting, namely, water splitting starts earlier in the absence of spacers. In addition, the electroconvection in the thin boundary layer is slightly reduced when spacers are used, which reduces the mixing of the solution and consequently the transport of salt ions. On the other hand, spacers promote convective transport of the electrolyte solution from the middle part of the channel with a high content of salt ions to the vicinity of the ion-exchange membranes, where the salt content is lower, and therefore increase the transport of salt ions.

## Data Availability

Data is contained within the article.
